# Peristaltic pump with heat and mass transfer of a fractional second grade fluid through porous medium inside a tube

**DOI:** 10.1038/s41598-022-14773-y

**Published:** 2022-06-23

**Authors:** A. M. Abd-Alla, S. M. Abo-Dahab, Esraa N. Thabet, M. A. Abdelhafez

**Affiliations:** 1 Department of Mathematics, Faculty of Science, Sohag University, Sohag, Egypt; 2 Department of Mathematics, Faculty of Science, South Valley University, Qena, Egypt; 3 Department of Computer Science, Faculty of Computers and Information, Luxor University, Luxor, Egypt

**Keywords:** Applied mathematics, Computational science

## Abstract

In magnetic resonance imaging (MRI), this MRI is used for the diagnosis of the brain. The dynamic of these particles occurs under the action of the peristaltic waves generated on the flexible walls of the brain. Studying such fluid flow of a Fractional Second-Grade under this action is therefore useful in treating tissues of cancer. This paper deals with a theoretical investigation of the interaction of heat and mass transfer in the peristaltic flow of a magnetic field fractional second-grade fluid through a tube, under the assumption of low Reynolds number and long-wavelength. The analytical solution to a problem is obtained by using Caputo's definition. The effect of different physical parameters, the material constant, magnetic field, and fractional parameter on the temperature, concentration, axial velocity, pressure gradient, pressure rise, friction forces, and coefficient of heat and mass transfer are discussed with particular emphasis. The computed results are presented in graphical form. It is because the nature of heat and mass transfer coefficient is oscillatory which is following the physical expectation due to the oscillatory nature of the tube wall. It is perceived that with an increase in Hartmann number, the velocity decreases. A suitable comparison has been made with the prior results in the literature as a limiting case of the considered problem.

## Introduction

Peristaltic flows of non-Newtonian fluids in the presence of a magnetic field have recently piqued attention in physiology, particularly in the form of a device known as Magnetic Resonance Imaging (MRI). This MRI is used to diagnose brain and vascular disorder, as well as the entire human body. In the description of viscoelastic properties, fractional calculus has had a lot of success. Vertical turbine pumps are a tried-and-true workhorse pump used in industrial applications all over the world. The oil and gas, chemical, petrochemical, desalination, power, and mining industries all benefit from this pump design. Vertical pumps are built to handle difficult apps and have a long history dating back to their invention in the Los Angeles more than a century ago. The effects of fractional Maxwell fluids on peristaltic with heat and mass transfer were investigated by Bayones et al.^1^. Moreover, the analytical solution for concentration, temperature, tangential stress, axial velocity, and coefficient of heat transfer was deduced. Hameed et al.^2^ discussed the peristaltic flow of the fractional second-grade fluid confined in a cylindrical tube. They found that an increase in the constant of fractional second-grade fluid results in a decrease in velocity profile for the case of fractional second-grade fluid whereas the velocity remains unchanged for the case of second-grade fluid. Under the consideration of long-wavelength, Haroun^3^ is devoted to the study of peristaltic transport of a fourth-grade fluid in an inclined asymmetric channel. Heat and mass transfer analysis in the mixed convective peristaltic flow of fourth-grade fluid under viscous dissipation with Dufour and Soret effects was debated by Mustafa et al.^4^ where the resulting coupled nonlinear boundary value problem (BVP) was solved numerically by using Keller–box method. Krishna et al.^5^ explored theoretically the Hall and Ion slip impacts on an unsteady laminar MHD convective rotating flow of heat-generating or absorbing second-grade fluid over a semi-infinite vertical moving permeable surface. Rasool and Wakif^6^ determined the impact of Cattaneo–Christov model and convective boundary on second-grade nanofluid flow alongside a Riga pattern. The governing nonlinear problem was converted into ordinary problems via suitably adjusted transformations and the Spectral local linearization method was incorporated to find the solutions to the nonlinear problems. VeeraKrishna and Reddy^7^ considered the transient MHD flow of a reactive second-grade fluid through a porous medium between two infinitely long horizontal parallel plates, and the transient momentum equations were solved analytically using the Laplace transform technique. Hayat et al.^8^ addressed the heat and mass transfer in the peristalsis of MHD third-grade material through curved configuration. Ali et al.^9^ displayed the peristaltic flow of a third-grade fluid in a circular cylindrical tube when the no-slip condition at the tube wall. They observed that an increase in the slip parameter decreases the peristaltic pumping rate for a given pressure rise. Tripathi^10^ was investigating the transportation of a viscoelastic fluid with a fractional second-grade model by peristalsis through a cylindrical tube under the assumptions of long-wavelength and low Reynolds number. Abd-Alla et al.^11^ described the effect of the endoscope and heat transfer on the peristaltic flow of the Jeffrey fluid across the distance between the uniform concentric tubes utilizing the assumption of the long-wavelength and low Reynolds number to approximate the governing equations of motion. Zhao^12^ exhibited the convection flow, the magnetic field, and velocity slip of a peristaltic motion of a fractional fluid. Javid et al.^13^ used the fourth-order Runge–Kutta method to analyze the interface of the thermal boundary layer, and the porosity effects were modeled using a Navier–Stokes equation with a body force term. Wahid et al.^14^ talked about the boundary layer flow and heat transfer on a viscoelastic fluid over a stretched surface in a porous medium with thermal radiation and viscous dissipation. Mainardi and Spada^15^ offered a comprehensive overview of fractional calculus-based viscoelastic models and investigated the basic fractional models in terms of creep, relaxation, and viscosity features. With the help of Hall and induced magnetic field effects, Singh and Vishwanath^16^ elucidated the convective flow of a viscoelastic electrically conducting fluid within an inclined channel boundary in a porous regime. The heat transfer and second-order slip impact on the MHD flow of fractional Maxwell fluid in a porous medium were explained by Amana et al.^17^. Also, Tripathi^18^ demonstrated the use of a fractional Maxwell model to investigate the peristaltic transport of viscoelastic non-Newtonian fluids in a channel. Under the influence of a uniform transverse magnetic field, Waghmode and Suneetha^19^ investigated the unsteady MHD rotating free convection flow of viscoelastic fluid through a porous media with simultaneous heat and mass transfer near an infinite vertical oscillating porous plate. Narla et al.^20^ used the fractional calculus approach to obtain an analytical solution for the flow of a viscoelastic fluid. The influences of fractional parameter, material constant, amplitude, and curvature parameter on the pressure and friction force across one wavelength are discussed numerically with the help of graphs. Tariq and Khan ^21^ revealed the behavior of second-grade dusty fluid flowing through a flexible tube whose walls are induced by the peristaltic movement implementing the regular perturbation technique to get the solutions. The electro-osmotic peristaltic flow of a viscoelastic fluid through a cylindrical micro-channel was studied by Guo and Qi^22^ where the analytical solutions of pressure gradient, stream function, and axial velocity were explored in terms of Mittag–Leffler function by Laplace transform method. Tripathi and Bég^23^ applied Caputo’s definition to determine approximate analytical solutions of inclined tube peristaltic flow of a fractional second-order biofluid. Imran et al.^24^ discovered the effect of heat and mass transfer on particle–fluid suspension for the Rabinowitsch fluid model with the stiffness and dynamic damping effects through Darcy–Brinkman–Forchheimer porous medium. Furthermore, Bayones et al.^25^ displayed the magnetized dissipative Soret of steady viscous incompressible two-dimensional Maxwell fluid flow in a porous medium over a stretching sheet with chemical reaction and Joule heating. Abd-Alla et al.^26^ deliberated the impacts of the gravitational forces, buoyancy forces, and magnetic field on velocity profiles, temperature, and concentration of the magneto-hydrodynamic peristalsis of Jeffery nanofluid through porous media. El-Dabe et al.^27^ found numerical solutions for the axial velocity, temperature, and nanoparticles concentration of a non-Newtonian nanofluid flow with heat transfer through a non-uniform inclined channel. Moreover, the effects of partial slip and the magnetic field on the peristaltic flow of Walter's B fluid through a porous medium were debated by Dabe et al.^28^.

With these motivations in mind, the present investigation aims to discuss the influence of the magnetic field on the peristaltic pump of a fractional second-grade fluid in a porous vertical tube. To our knowledge, a magnetic field in the peristaltic pump of a fractional second-grade fluid is still unexplored. The nonlinear equations of viscoelastic fluid with fractional second-grade fluid are simplified using the assumptions of long-wavelength and low Reynolds number and then the resulting equations have been solved analytically and numerically. Comparisons of both the solutions are also made. In the end, the graphical results against different physical parameters have also been presented and discussed.

## Caputo's definition

Caputo's definition of the fractional-order derivative is defined as ^10^:

1 $$ D^{{\alpha_{1} }} f(t) = \frac{1}{{\Gamma (1 - \alpha_{1} )}}\frac{d}{dt}\int_{i}^{t} {\frac{{f^{n} (\xi )}}{{(t - \xi )^{{\alpha_{1} + 1 - n}} }}} d\xi ,\,\,\,\,\,\,\,\,\,\,\left( {n - 1,\,\,{\text{Re}} (\alpha_{1} ) \le n,\,\,n \in N} \right)\, $$

where, $\alpha_{1}$ is the order of the derivative and is allowed to be real or even complex and $i$ is the initial value of function $f.$ For the Caputo's derivatives we have:

2 $$ D^{{\alpha_{1} }} t^{{\beta_{1} }} = \left\{ \begin{array}{ll} 0&\quad \left( {\beta_{1} \le \alpha_{1} - 1} \right) \hfill \\ \frac{{\Gamma (\beta_{1} + 1)}}{{\Gamma (\beta_{1} - \alpha_{1} + 1)}}\,t^{{\beta_{1} - \alpha }}&\quad \left( {\beta_{1} > \alpha_{1} - 1} \right) \\ \end{array} \right. $$

## Formulation of the problem

The constitutive equation for viscoelastic fluid with a fractional second-grade model is given by the following relation:

3 $$ \overline{S} = (1 + \overline{{\lambda_{1} }}^{{\alpha_{1} }} \frac{\partial }{{\partial \overline{t} }})\mathop \gamma \limits^{.} . $$

Also, note that $D_{{\overline{t} }}^{{\alpha_{1} }} = \partial_{{\overline{t} }}^{{\alpha_{1} }}$, denoting the fractional differentiation operator of order $\alpha_{1}$ concerning $\overline{t} .$

**Figure 1:**
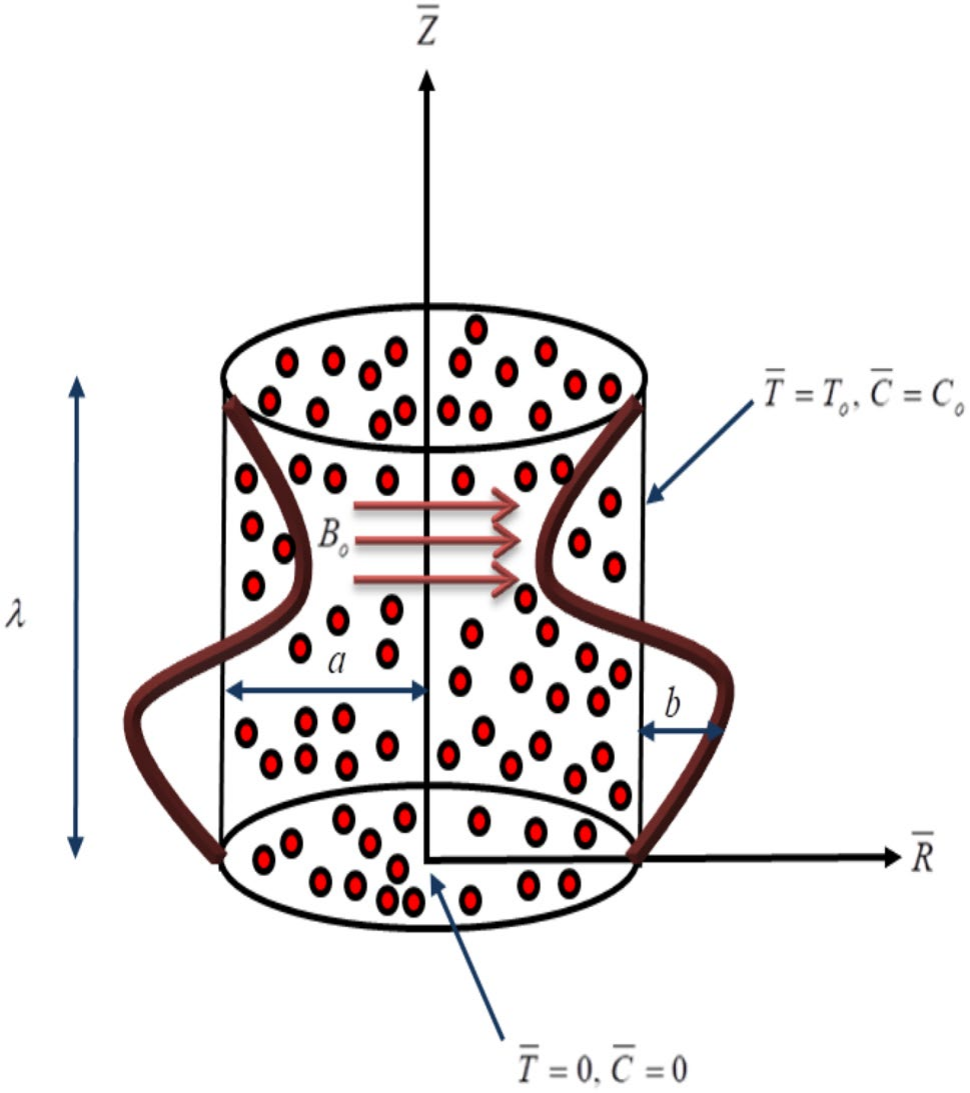
Schematic diagram of the physical model.

Let us consider a fractional second-grade fluid through a porous vertical tube. In the axisymmetric cylindrical polar coordinate system $(R,Z)$, $R - axis$ is the radial coordinate and *Z*-axis is the coordinate along the axes of the tube see Fig. 1. The geometry of the tube wall is mathematically given by:

4 $$ \overline{H} = a + b\,\cos^{2} \left( {\frac{\pi }{\lambda }\left( {\overline{Z} - c\overline{t} } \right)} \right)\,. $$

The equations governing the flow of viscoelastic fluid with a fractional second-grade model for axisymmetric flows in the fixed frame are^10^:

5 $$ \frac{1}{{\overline{R} }}\frac{\partial }{{\partial \overline{R} }}\left( {\overline{R} \overline{U} } \right) + \frac{{\partial \overline{W} }}{{\partial \overline{Z} }} = 0. $$

6 $$ \rho \left[ {\frac{{\partial \overline{U} }}{{\partial \overline{t} }} + \overline{U} \frac{{\partial \overline{U} }}{{\partial \overline{R} }} + \overline{W} \frac{{\partial \overline{U} }}{{\partial \overline{Z} }}} \right] = - \frac{{\partial \overline{P} }}{{\partial \overline{R} }} + \mu \left( {1 + \overline{{\lambda_{1} }}^{{\alpha_{1} }} \overline{D}_{{\overline{t} }}^{{\alpha_{1} }} } \right)\,\left[ {\frac{\partial }{{\partial \overline{R} }}\left( {\frac{1}{{\overline{R} }}\frac{\partial }{{\partial \overline{R} }}\left( {\overline{R} \overline{U} } \right) + \frac{{\partial^{2} \overline{U} }}{{\partial \overline{Z}^{2} }}} \right)} \right] - \frac{\mu }{{k_{1} }}\overline{U} , $$

7 $$ \begin{aligned} \rho \left[ {\frac{{\partial \overline{W} }}{{\partial \overline{t} }} + \overline{U} \frac{{\partial \overline{W} }}{{\partial \overline{R} }} + \overline{W} \frac{{\partial \overline{W} }}{{\partial \overline{Z} }}} \right] &= - \frac{{\partial \overline{P} }}{{\partial \overline{Z} }} + \mu \left( {1 + \overline{{\lambda_{1} }}^{{\alpha_{1} }} \overline{D}_{{\overline{t} }}^{{\alpha_{1} }} } \right)\,\left[ {\frac{1}{{\overline{R} }}\frac{\partial }{{\partial \overline{R} }}\left( {\overline{R} \frac{{\partial \overline{W} }}{{\partial \overline{R} }}} \right) + \frac{{\partial^{2} \overline{W} }}{{\partial \overline{Z}^{2} }}} \right]   \\ &\quad+ \rho g\overline{{\alpha_{t} }} \left( {\overline{T} - T_{0} } \right) + \rho g\overline{{\alpha_{c} }} \left( {\overline{C} - C_{0} } \right) - \sigma B_{o} \overline{W} - \frac{\mu }{{k_{1} }}\overline{W} , \end{aligned} $$

8 $$ \rho_{Cp} \left[ {\frac{{\partial \overline{T} }}{{\partial \overline{t} }} + \overline{U} \frac{{\partial \overline{T} }}{{\partial \overline{R} }} + \overline{W} \frac{{\partial \overline{T} }}{{\partial \overline{Z} }}} \right] = K\left( {\frac{{\partial^{2} \overline{T} }}{{\partial \overline{R}^{2} }} + \frac{1}{{\overline{R} }}\frac{{\partial \overline{T} }}{{\partial \overline{R} }} + \frac{{\partial^{2} \overline{T} }}{{\partial \overline{Z}^{2} }}} \right) + Q_{o} , $$

9 $$ \left[ {\frac{{\partial \overline{C} }}{{\partial \overline{t} }} + \overline{U} \frac{{\partial \overline{C} }}{{\partial \overline{R} }} + \overline{W} \frac{{\partial \overline{C} }}{{\partial \overline{Z} }}} \right] = D_{m} \left( {\frac{{\partial^{2} \overline{C} }}{{\partial \overline{R}^{2} }} + \frac{1}{{\overline{R} }}\frac{{\partial \overline{C} }}{{\partial \overline{R} }} + \frac{{\partial^{2} \overline{C} }}{{\partial \overline{Z}^{2} }}} \right) + \frac{{D_{m} K_{T} }}{{T_{m} }}\left( {\frac{{\partial^{2} \overline{T} }}{{\partial \overline{R}^{2} }} + \frac{1}{{\overline{R} }}\frac{{\partial \overline{T} }}{{\partial \overline{R} }} + \frac{{\partial^{2} \overline{T} }}{{\partial \overline{Z}^{2} }}} \right). $$

Using the transformations mentioned below to associate the moving $(\overline{r} ,\overline{z} )$ and fixed frames $(\overline{R} ,\overline{Z} ).$

10 $$ \overline{r} = \overline{R},\,\,\overline{z} = \overline{Z} + c\overline{t},\,\,\,\overline{u} = \overline{U},\;\overline{w} = \overline{W} - c,\,\,\overline{p} = \overline{P},\,\,\overline{T} = T,\,\,\overline{C} = C,\,\,q = F - h^{2} . $$

The boundary conditions for the flow are defined as follows:

11a $$ \frac{{\partial \overline{W} }}{{\partial \overline{R} }} = 0\,\,\,\,\,at\,\,\,\,\overline{R} = 0,\,\,\,\,\,\,\,\,\overline{W} = 0\,\,\,\,\,at\,\,\,\,\,\overline{R} = \overline{H} , $$

11b $$ \frac{{\partial \overline{T} }}{{\partial \overline{R} }} = 0\,\,\,\,\,at\,\,\,\,\overline{R} = 0,\,\,\,\,\,\,\,\,\overline{\theta } = 0\,\,\,\,\,at\,\,\,\,\,\overline{R} = \overline{H} , $$

11c $$ \frac{{\partial \overline{C} }}{{\partial \overline{R} }} = 0\,\,\,\,\,at\,\,\,\,\overline{R} = 0,\,\,\,\,\,\,\,\,\overline{\Theta } = 0\,\,\,\,\,at\,\,\,\,\,\overline{R} = \overline{H} . $$

Taking into account the dimensionless quantities as follow:

12 $$ \begin{gathered} r = \frac{{\overline{R}}}{a},\,\,z = \frac{{\overline{z}}}{\lambda },\,\,u = \frac{{\overline{u}}}{c}\delta ,\,\,w = \frac{{\overline{w}}}{c},\,\,t = \frac{{c\overline{t}}}{\lambda },\,\,h = \frac{{\overline{H}}}{a},\,\,\phi = \frac{b}{a}, \hfill \\ \delta = \frac{a}{\lambda },\,\,p = \frac{{a^{2} \overline{p}}}{c\lambda \mu },\,M = \sqrt {\frac{\sigma }{\mu }} B_{0} a,\,\,\lambda_{1} = \frac{{c\overline{\lambda }_{1} }}{\lambda },\,\,\Pr = \frac{{\mu c_{p} }}{K},\,\,{\text{Re}} = \frac{ca}{\upsilon },\,\,S = \frac{{\overline{S}a}}{\mu c}, \hfill \\ Gr = \frac{{\rho g\overline{{\alpha_{t} }} T_{o} a^{2} }}{\mu c},\,\,Gn = \frac{{\rho g\overline{{\alpha_{c} }} C_{o} a^{2} }}{\mu c},\,\,\,\theta = \frac{{\overline{T} - T_{0} }}{{T_{o} }},\,\,\,\Theta = \frac{{\overline{C} - C_{0} }}{{C_{0} }}, \hfill \\ Sc = \frac{\mu }{{D_{m} \rho }},\,\,Sr = \frac{{\rho D_{m} K_{T} T_{o} }}{{\mu T_{m} C_{o} }},\,\,\beta = \frac{{a^{2} Q_{o} }}{{KT_{o} }}. \hfill \\ \end{gathered} $$

## Solution of the problem

The preceding equations are simplified to the following when the above-mentioned adjustments and non-dimensional variables (12) are taken into account:

13 $$ \frac{1}{r}\frac{\partial }{\partial r}\left( {ru} \right) + \frac{\partial w}{{\partial z}} = 0. $$

14 $$ {\text{Re}} \delta^{3} \left[ {u\frac{\partial u}{{\partial r}} + \left( {w + 1} \right)\frac{\partial u}{{\partial z}}} \right] = - \frac{\partial p}{{\partial r}} + \delta^{2} \left( {1 + \lambda_{1}^{{\alpha_{1} }} \frac{{\partial^{{\alpha_{1} }} }}{{\partial t^{{\alpha_{1} }} }}} \right)\left[ {\frac{\partial }{\partial r}\left( {\frac{1}{r}\frac{\partial }{\partial r}\left( {ru} \right)} \right) + \delta^{2} \frac{{\partial^{2} u}}{{\partial z^{2} }}} \right] - \frac{{\delta^{2} }}{Da}u, $$

15 $$ \begin{aligned} {\text{Re}} \delta \left[ {u\frac{\partial w}{{\partial r}} + \left( {w + 1} \right)\frac{\partial w}{{\partial z}}} \right] &= - \frac{\partial p}{{\partial z}} + \left( {1 + \lambda_{1}^{{\alpha_{1} }} \frac{{\partial^{{\alpha_{1} }} }}{{\partial t^{{\alpha_{1} }} }}} \right)\left[ {\frac{1}{r}\frac{\partial }{\partial r}\left( {r\frac{\partial w}{{\partial r}}} \right) + \delta^{2} \frac{{\partial^{2} w}}{{\partial z^{2} }}} \right] + Gr\theta \\ &\quad \quad \quad+ Gn\Theta - \left( {M^{2} + \frac{1}{Da}} \right)\left( {w + 1} \right),  \end{aligned} $$

16 $$ {\text{Re}} \Pr \delta \left[ {u\frac{\partial \theta }{{\partial r}} + \left( {w + 1} \right)\frac{\partial \theta }{{\partial z}}} \right] = \left( {\frac{{\partial^{2} \theta }}{{\partial r^{2} }} + \frac{1}{r}\frac{\partial \theta }{{\partial r}} + \delta^{2} \frac{{\partial^{2} \theta }}{{\partial z^{2} }}} \right) + \beta , $$

17 $$ {\text{Re}} \delta \left[ {u\frac{\partial \Theta }{{\partial r}} + \left( {w + 1} \right)\frac{\partial \Theta }{{\partial z}}} \right] = \frac{1}{Sc}\left( {\frac{{\partial^{2} \Theta }}{{\partial r^{2} }} + \frac{1}{r}\frac{\partial \Theta }{{\partial r}} + \delta^{2} \frac{{\partial^{2} \Theta }}{{\partial z^{2} }}} \right) + Sr\left( {\frac{{\partial^{2} \theta }}{{\partial r^{2} }} + \frac{1}{r}\frac{\partial \theta }{{\partial r}} + \delta^{2} \frac{{\partial^{2} \theta }}{{\partial z^{2} }}} \right)\,. $$

## Analytical solution

Furthermore, the hypothesis of the long-wavelength approach is also supposed. Now, $\delta$ is very small so that it can be tended to zero. Thus, the $\delta < < 1$ dimensionless governing Eqs. (14), (15), (16), and (17) by using this hypothesis, may be written as:

18 $$ 0 = - \frac{\partial p}{{\partial r}}, $$

19 $$ 0 = - \frac{dp}{{dz}} + \left( {1 + \lambda_{1}^{{\alpha_{1} }} \frac{{\partial^{{\alpha_{1} }} }}{{\partial t^{{\alpha_{1} }} }}} \right)\left[ {\frac{1}{r}\frac{\partial }{\partial r}\left( {r\frac{\partial w}{{\partial r}}} \right)} \right] + Gr\theta + Gn\Theta - \left( {M^{2} + \frac{1}{Da}} \right)\left( {w + 1} \right), $$

20 $$ 0 = \frac{{\partial^{2} \theta }}{{\partial r^{2} }} + \frac{1}{r}\frac{\partial \theta }{{\partial r}} + \beta , $$

21 $$ 0 = \frac{1}{Sc}\left( {\frac{{\partial^{2} \Theta }}{{\partial r^{2} }} + \frac{1}{r}\frac{\partial \Theta }{{\partial r}}} \right) + Sr\left( {\frac{{\partial^{2} \theta }}{{\partial r^{2} }} + \frac{1}{r}\frac{\partial \theta }{{\partial r}}} \right)\,. $$

The relevant boundary conditions are:

22a $$ \frac{\partial w}{{\partial r}} = 0\,\,\,\,\,at\,\,\,\,r = 0,\,\,\,\,\,\,\,\,w = 0\,\,\,\,\,at\,\,\,\,\,r = h = 1 + \varphi \cos^{2} \left( {\pi \,z} \right), $$

22b $$ \frac{\partial \theta }{{\partial r}} = 0\,\,\,\,\,at\,\,\,\,r = 0,\,\,\,\,\,\,\,\,\theta = 0\,\,\,\,\,at\,\,\,\,\,r = h = 1 + \varphi \cos^{2} \left( {\pi \,z} \right), $$

22c $$ \frac{\partial \Theta }{{\partial r}} = 0\,\,\,\,\,at\,\,\,\,r = 0,\,\,\,\,\,\,\,\,\Theta = 0\,\,\,\,\,at\,\,\,\,\,r = h = 1 + \varphi \cos^{2} \left( {\pi \,z} \right). $$

Equation (18) specifies that $p$ is only a function of $z$.

The non-dimensional formulas for volume flow rate $F,$ pressure rise $\Delta P_{\lambda }$, and frictional forces $F_{\lambda }$ are generated by the following equations:

23 $$ F = \int\limits_{0}^{h} {2rw} dr $$

24 $$ \Delta P_{\lambda } = \int\limits_{0}^{1} {\left(\frac{dp}{{dx}}\right)dx} $$

25 $$ F_{\lambda } = \int\limits_{0}^{1} {h\left( - \frac{dp}{{dx}}\right)dx} $$

The average of the volume flow rate $Q$ along one time period gives:

26 $$ Q = \int\limits_{0}^{1} F dt = \, \int\limits_{0}^{1} {\left( {q + h^{2} } \right)} dt = q + 1 - \varphi + \frac{3}{8}\varphi^{2} . $$

Temperature, concentration and axial velocity solutions can be described as follows:

27 $$ \theta = \frac{\beta }{4}\left( {h^{2} - r^{2} } \right), $$

28 $$ \Theta = \frac{SrSc\beta }{4}\left( {r^{2} - h^{2} } \right), $$

29 $$ w = \frac{{\left( \begin{gathered} A\left( {A + \frac{dp}{{dz}}} \right) + Gn\left( {4f + A\left( { - h^{2} + r^{2} } \right)} \right)Sr\,Sc\,\beta - 2Gr\left( {4f + A\left( { - h^{2} + r^{2} } \right)} \right)B + \hfill \\ \left( {8\left( {A^{2} + A\frac{dp}{{dz}} - 4f\,Gn\,Sr\,Sc + f\,Gr\,\beta } \right)\,oF_{1} \left( { - ;\,1;\frac{{A\,r^{2} }}{4f}} \right)} \right) \hfill \\ \end{gathered} \right)}}{{oF_{1} \left( { - ;\,1;\frac{{Ah^{2} }}{4f}} \right)}}. $$

From Eqs. (23), (26), we can deduce:

30 $$ \frac{dp}{{dz}} = \frac{{\left( {Q - a_{1} - a_{2} } \right)}}{{a_{3} }}. $$

where, the constants and $a_{i} \,,\,\,i = 1:3,$ $f,A,B$ are given in Appendix S1.

The heat transfer coefficient is indicated as follows:

31 $$ Zh = \left[ {\frac{\partial \theta }{{\partial r}} \times \frac{\partial r}{{\partial z}}} \right]_{r = h} . $$

So, the solution of heat transfer is given by:

32 $$ Zh = \frac{\beta \pi \varphi h}{2}\,\sin \left( {2\pi \,z} \right). $$

The mass transfer coefficient is indicated as follows:

33 $$ Zm = \left[ {\frac{\partial \Theta }{{\partial r}} \times \frac{\partial r}{{\partial z}}} \right]_{r = h} . $$

So, the solution of heat transfer is given by:

34 $$ Zm = - \frac{Sr\,Sc\beta \pi \varphi h}{2}\,\sin \left( {2\pi \,z} \right). $$

## Numerical results and discussion

After getting analytical solutions of Eqs. (19–21) about the conditions Eqs. (22a–22c), and using Matlab software, the central purpose is to compute the outcomes of significant parameters relevant to the considered mathematical problem. The impacts of these parameters that affect different flow profiles such as the temperature $\theta ,$ concentration $\Theta ,$ axial velocity $w,$ pressure gradient $\frac{dp}{{dz}},$ pressure rise $\Delta p_{\lambda } ,$ friction forces $F_{\lambda } ,$ coefficient of heat and mass transfer $Zh\,\,and\,\,Zm$ in the porous vertical tube are carefully analyzed and sketched in Figures “2–10” for different models of fractional second-grade fluid, second-grade fluid, and classical Navier–Stokes fluid, respectively.

**Figure 2:**
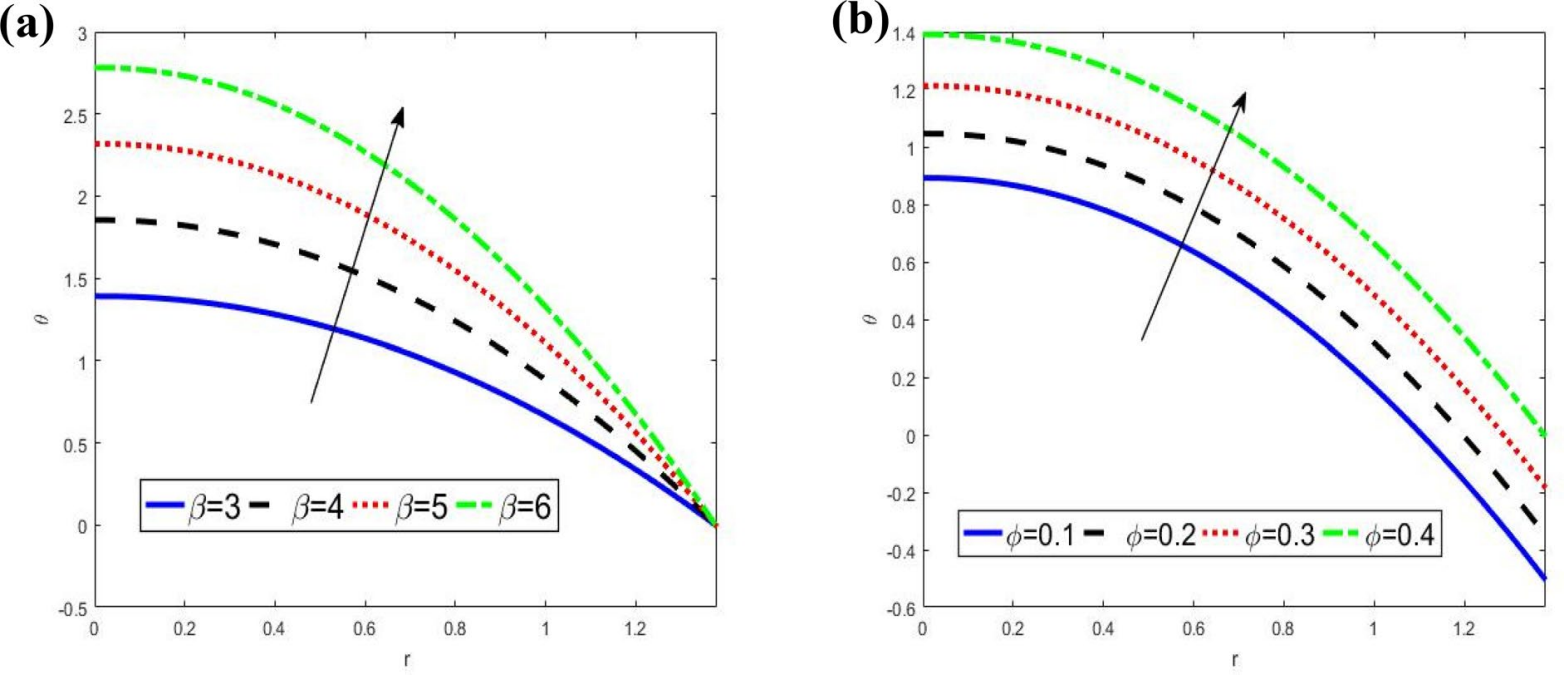
Analysis of temperature $$\theta .$$ (**a**) Analysis of $$\theta$$ against $$\beta .$$ (**b**) Analysis of $$\theta$$ against $$\varphi .$$ For the values $$z = 0.1,\,$$$$\varphi = 0.4,$$ $$\beta = 3$$.

It can be observed clearly from Fig. 2 that temperature $\theta$ increases with an increase in the heat source / sink parameter $\beta$ and wave amplitude $\varphi$ at the inlet as well as downstream, while it decreases with increasing of the radial $r$. The maximum temperature at inlet and downstream is obtained for different values $\beta$ and $\varphi$ respectively. Also, the temperature satisfied the boundary conditions.

**Figure 3:**
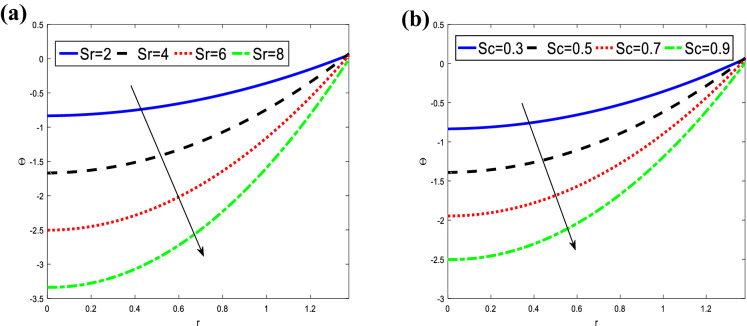
Analysis of concentration $\Theta .$ (**a**) Analysis of $\Theta$ against $Sr.$ (**b**) Analysis of $\Theta$ against $Sr.$ For the values $z = 0.1,\,\,\,\varphi = 0.4,\,\,\beta = 3,\,\,Sr = 2,\,\,Sc = 0.3$.

Figure 3 indicates the variations of the concentration $\Theta$ concerning the radial $r$ for different values of Soret number $Sr$ and Schmidt number $Sc$. It is observed that the concentration decreases with an increase in the Soret number and Schmidt number at the inlet as well as downstream, while it increases with increasing of the radial $r$. The maximum concentration at the inlet and downstream is obtained for different values $Sr$ and $Sc,$ respectively. It is observed that the concentration satisfied the boundary conditions.

**Figure 4:**
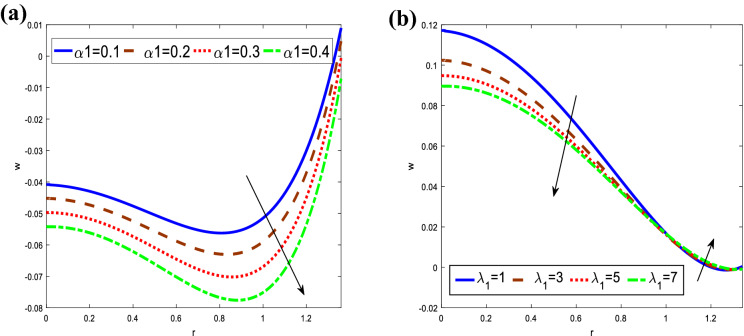
Analysis of axial velocity $w.$ (**a**) Analysis of $w$ against $\alpha_{1} .$ (**b**) Analysis of $\Theta$ against $Sr.$ For the values $z = 0.1,\,\,\,\varphi = 0.4,\,\,\beta = 3,\,\,Sc = 0.3,\,\,$ $\,Sr = 2,\,\,\alpha_{1} = 0.4,\,\,t = 0.2,\,\,Gr = 3,\,\,Gn = 3,\,\,M = 0.1,\,\,Da = 1$.

The findings are shown in Fig. 4 elucidate the variations of the axial velocity $w$ concerning the radial $r$ for different values of fractional time derivative parameter $\alpha_{1} ,$ and the ratio of relaxation to retardation times $\lambda_{1}$. It is observed that the axial velocity decreases with an increase in the fractional time derivative parameter in the whole range of $r,$ while it decreases with increasing the ratio of relaxation to retardation times in the interval $0 \le r \le 1,$ as well as it increases in the interval $1 \le r \le 1.36$. One can observe that axial velocity is in oscillatory behavior, which may be due to peristalsis. In addition, the axial velocity satisfied the boundary conditions.

**Figure 5:**
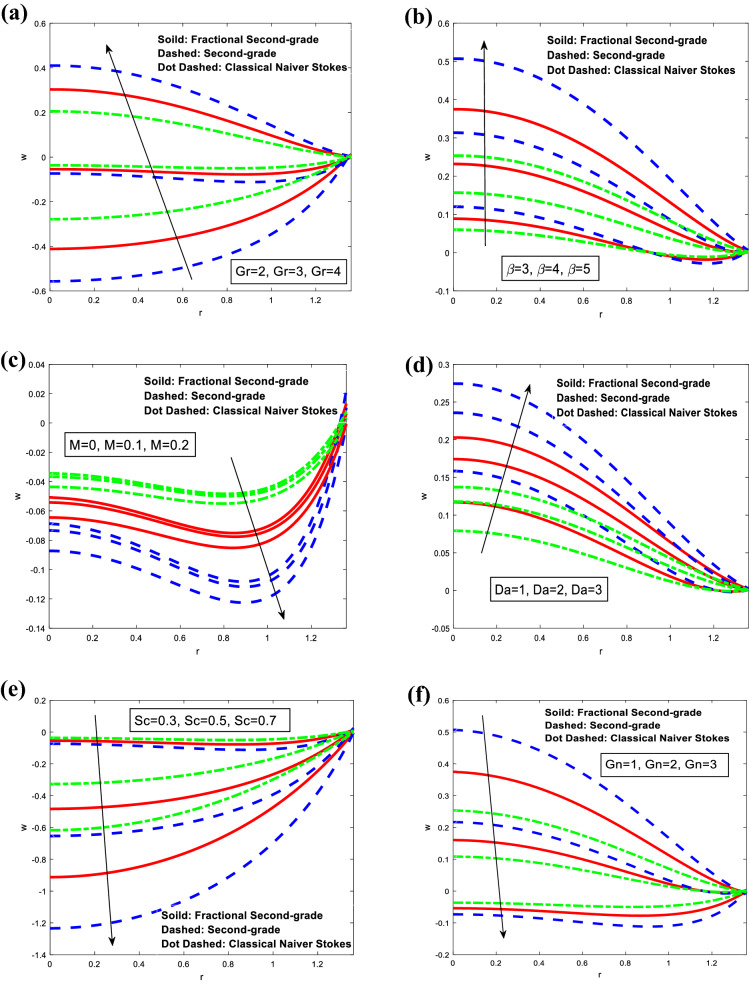
Analysis of axial velocity $w$ in different modules. (**a**) Analysis of $w$ against $Gr.$ (**b**) Analysis of $w$ against $\beta .$ (**c**) Analysis of $w$ against $M.$ (**e**) Analysis of $w$ against $Da.$(**d**) Analysis of $w$ against $Sc.$ (**f**) Analysis of $w$ against $Gn.$ For the values $z = 0.1,\,\,\,\varphi = 0.4,\,\,\alpha_{1} = 0.4,\,\,Sc = 0.3,\,\,Sr = 2,\,\,\lambda_{1} = 1,\,\,t = 0.2,\,\,Gr = 3,\,\,Gn = 3,\,\,M = 0.1\,,\beta = 3,\,\,Da = 1$.

The variations of the axial velocity *w* concerning the radial *r* for different models are plotted in Fig. 5. It is remarkable from this figure that an increment in Grashof number $Gr,$ heat source/sink parameter $\beta ,$ and Darcy number $Da$ results in a significant increase in the axial velocity. The converse behavior is noted for Hartman number $M,$ and Schmidt number $Sc.$ i.e. the axial velocity is reduced by increasing Hartman number, and Schmidt number for different models of classical Navier–Stokes fluid, fractional second-grade fluid, and second-grade fluid respectively. For Local concentration Grashof number, the axial velocity was also reduced by increasing it for different models of second-grade fluid, fractional second-grade fluid, and classical Navier–Stokes fluid, respectively. Furthermore, it satisfied the boundary conditions.

**Figure 6:**
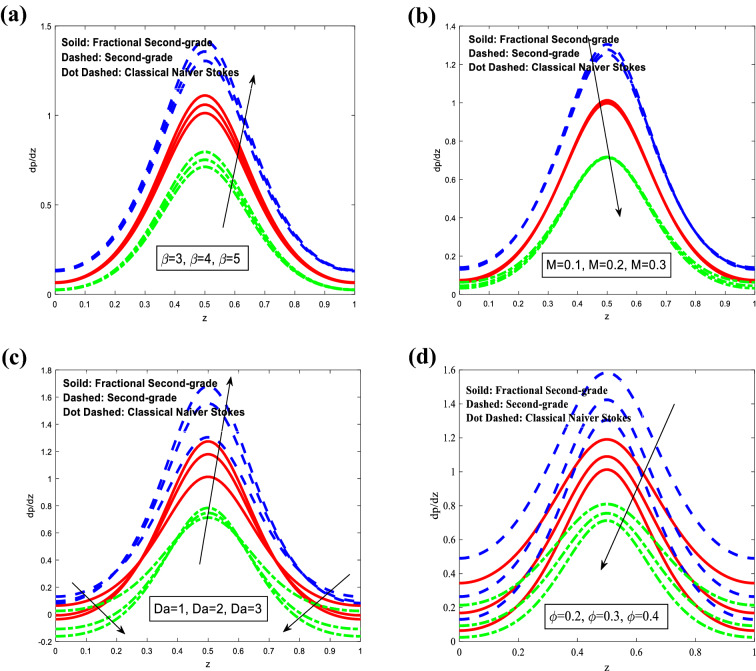
Analysis of the axial pressure gradient $\frac{dp}{{dz}}$ in different modules. (**a**) Analysis of $\frac{dp}{{dz}}$ against $\beta .$ (**b**) Analysis of $\frac{dp}{{dz}}$ against $M.$ (**c**) Analysis of $\frac{dp}{{dz}}$ against $Da.$ (**d**) Analysis of $\frac{dp}{{dz}}$ against $\varphi .$ For the values $z = 0.1,\,\,\,\varphi = 0.4,\,\,\alpha_{1} = 0.4,\,\,Sc = 0.3,\,\,Sr = 2,\,\,\lambda_{1} = 1,\,\,t = 0.2,\,\,Gr = 3,\,\,Gn = 3,\,\,M = 0.1\,,\beta = 3,\,\,Da = 1$.

Figure 6 depicts the behavior of variations of the axial pressure gradient $\frac{dp}{{dz}}$ concerning the *z*-axis for different values of heat source/sink parameter *β* Hartman number *M* Darcy number $Da$ and wave amplitude $\varphi$ respectively. It is observed that the axial pressure gradient increases with an increase in the heat source/sink parameter. For Darcy number, it has oscillated behavior in the whole range *z*-axis, while it decreases with increasing of Hartman number and wave amplitude. All these previous behaviors for different models of second-grade fluid, fractional second-grade fluid, and classical Navier–Stokes fluid, respectively.

**Figure 7:**
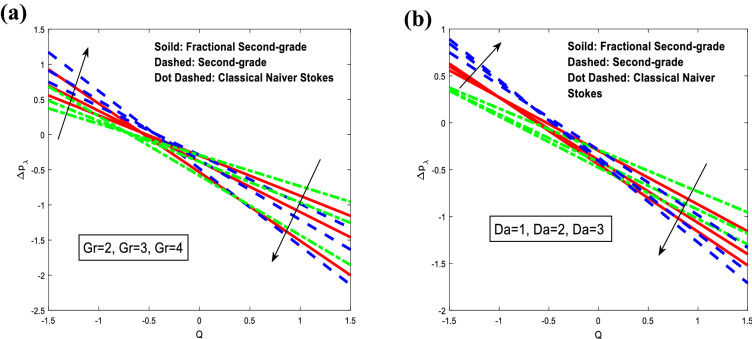
Analysis of pressure rise $\Delta p_{\lambda }$ in different modules. (**a**) Analysis of $\Delta p_{\lambda }$ against $Gr.$ (**b**) Analysis of $\Delta p_{\lambda }$ against $Da.$ For the values $z = 0.1,\,\,\,\varphi = 0.4,\,\,\beta = 3,\,\,Sc = 0.3,\,\,Sr = 2,\,\,\alpha_{1} = 0.4,\,\,t = 0.2,\,\,Gr = 3,\,\,Gn = 3,\,\,M = 0.1,\,\,\,Da = 1,\,\,\lambda_{1} = 1.$.

The impacts of parameters Grashof number $Gr$ and Darcy number $Da$ on the pressure rise $\Delta p_{\lambda }$ versus the mean flow rate $Q$ are demonstrated in Fig. 7. For the classical Navier–Stokes fluid model, the pressure rise increases rapidly with the increase of Grashof number when $Q \in ( - 1.5, - 0.65),$ and it decreases rapidly when $Q \in ( - 0.65,1.5).$ whereas, the pressure rise decreases rapidly with the increase of Darcy number when $Q \in ( - 1.5,1.5).$ For fractional second-grade fluid model, it is also observed that the pressure rise increases rapidly with the increase of Grashof number when $Q \in ( - 1.5, - 0.55),$ and it decreases rapidly when $Q \in ( - 0.55,1.5).$ Although, it increases rapidly with the increase of Darcy number when $Q \in ( - 1.5, - 1),$ and decreases with the increase of Darcy number when $Q \in ( - 1,1.5).$ For second-grade fluid model, it is seen that the pressure rise increases rapidly with the increase of Grashof number when $Q \in ( - 1.5, - 0.45),$ and it decreases rapidly when $Q \in ( - 0.45,1.5).$ However, it increases rapidly with the increase of Darcy number when $Q \in ( - 1.5, - 0.6),$ and decreases with the increase of Darcy number when $Q \in ( - 0.6,1.5).$ As expected, the pressure rise results in higher values for small mean volume flow rates and lower values for large $Q$. Furthermore, peristaltic pumping takes place in this area $- 1.5 \le Q \le 1.5,$ otherwise augmented pumping occurs.

**Figure 8:**
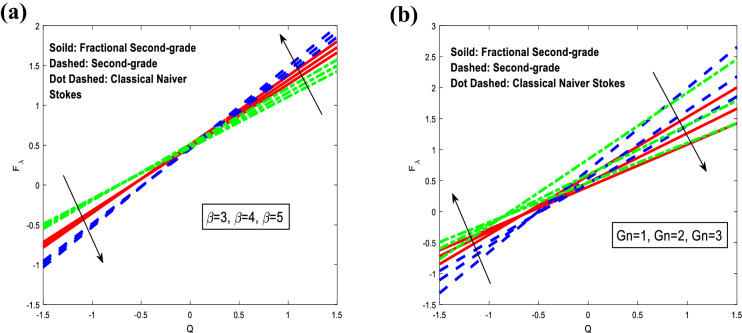
Analysis of friction forces $F_{\lambda }$ in different modules. (**a**) Analysis of $F_{\lambda }$ against $\beta .$ (**b**) Analysis of $F_{\lambda }$ against $Gn.$ For the values $z = 0.1,\,\,\,\varphi = 0.4,\,\,\beta = 3,\,\,Sc = 0.3,\,\,Sr = 2,\,\,\lambda_{1} = 1,\,\,t = 0.2,\,\,Gr = 3,\,\,\alpha_{1} = 0.4,\,\,M = 0.1,\,\,\beta = 3,\,\,\alpha_{1} = 0.4.$.

Figure 8 is schemed to check how the friction forces $F_{\lambda }$ is affected with the variations of heat source/sink $\beta$ and Local concentration Grashof number $Gn.$ For classical Navier–Stokes fluid model, the friction forces decreases rapidly with the increase of heat source/sink parameter when $Q \in ( - 1.5, - 0.65),$ and it increases rapidly when $Q \in ( - 0.65,1.5).$ while, the friction forces increases rapidly with the increase of Local concentration Grashof number when $Q \in ( - 1.5, - 0.85),$ and decreases rapidly when $Q \in ( - 0.85,1.5).$ For fractional second-grade fluid model, It is also observed that the friction forces decreases rapidly with the increase of heat source/sink parameter when $Q \in ( - 1.5, - 0.5),$ and it increases rapidly when $Q \in ( - 0.5,1.5).$ Although, it increases rapidly with the increase of Local concentration Grashof number when $Q \in ( - 1.5, - 0.7),$ and decreases with the increase of it when $Q \in ( - 0.7,1.5).$ For second-grade fluid model, It is notice that the friction forces decreases rapidly with the increase of heat source/sink parameter when $Q \in ( - 1.5, - 0.5),$ and it decreases rapidly when $Q \in ( - 0.5,1.5).$ Nevertheless, it increases rapidly with the increase of Local concentration Grashof number when $Q \in ( - 1.5, - 0.6),$ and decreases with the increase of Darcy number when $Q \in ( - 0.6,1.5).$ As expected, the friction forces results in higher values for small volume flow rates and lower values for large *Q*. Furthermore, peristaltic pumping takes place in this area otherwise $- 1.5 \le Q \le 1.5,$ augmented pumping occurs.

**Figure 9:**
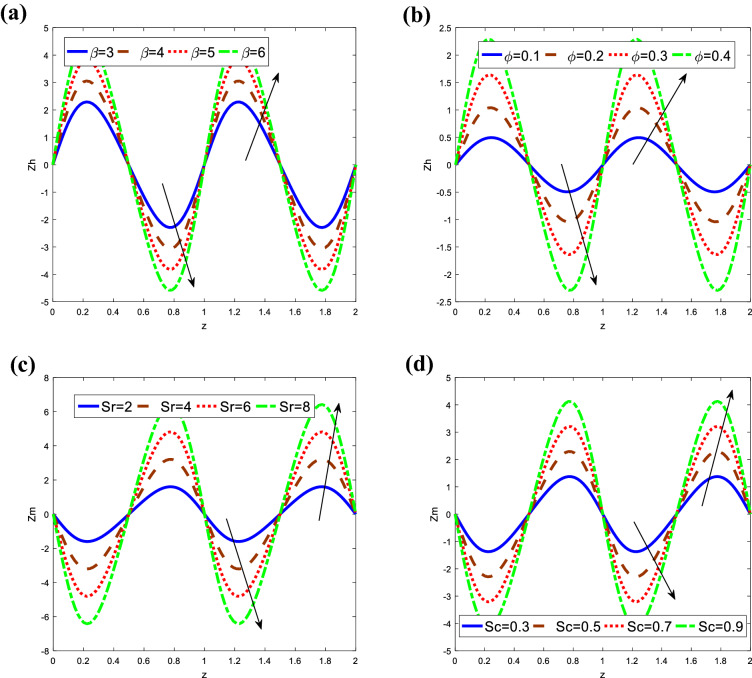
Analysis of coefficient of heat and mass transfer $Zh\,\,and\,\,Zm,$ respectively in different modules. (**a**) Analysis of $Zh\,$ against $\beta .$ (**b**) Analysis of $Zh\,$ against $\varphi .$ (**c**) Analysis of $Zm$ against $Sr.$ (**d**) Analysis of $Zm$ against $Sc.$ For the values $z = 0.1,\,\,Da = 1,\,\,\beta = 3,\,\,Sc = 0.3,\,\,\phi = 0.4,\,\,\alpha_{1} = 0.4,\,\,t = 0.2,\,\,Gr = 3,\,\,\alpha_{1} = 0.4,\,\,\,Sr = 2,\,\,M = 0.1,\,\,\lambda_{1} = 1$.

Figure 9 represents the behavior of heat and mass transfer coefficients at the wall of the tube. Heat and mass transfer coefficients have an oscillatory behavior due to peristalsis. It is obvious that the heat transfer coefficient $Zh$ increases and decreases with increasing of $\beta$ and $\varphi .$ while the mass transfer coefficient $Zm$ decreases and increases with increasing of $Sr$ and $Sc.$ Obviously, the increase in $\beta .$ $\varphi ,$ $Sr$ and $Sc$ increase in the amplitude of the heat and mass transfer coefficient in the whole range $z.$ From Fig. 9, one can observe that heat and mass transfer coefficient is an oscillatory behavior in the whole range, which may be due to peristalsis. When compared to the heat transfer coefficient, the mass transfer coefficient has the opposite behavior.

**Figure 10:**
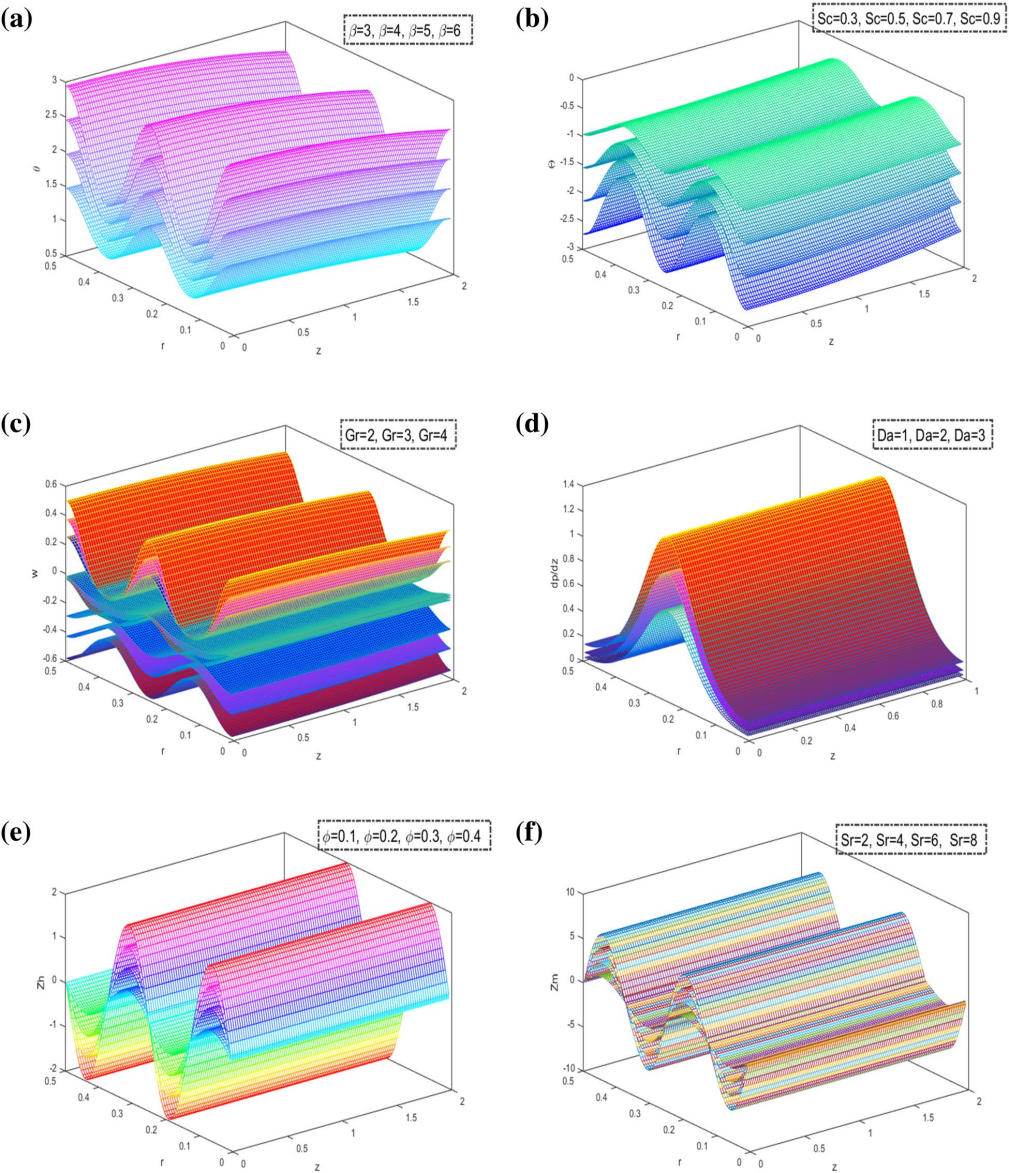
(**a**) 3D Analysis of temperature $\theta .$ (**b**) 3D Analysis of concentration $\Theta \,.$ (**c**) 3D Analysis of axial velocity $w\,.$ (**d**) 3D Analysis of axial pressure gradient $\frac{dp}{{dz}}\,.$ (**e**) 3D Analysis of coefficient of heat transfer $Zh\,.$ (**f**) 3D Analysis of coefficient of mass transfer $Zm$.

Figure 10 is plotted in $3D$ schematics concerning the temperature $\theta ,$ the concentration $\Theta ,$ the axial velocity $w,$ axial pressure gradient $\frac{dp}{{dz}}$ and the heat and mass transfer coefficient $Zh$ and $Zm$ concerning $r$ a $z$ xes in the presence of $\beta ,\,Sc,\,Gr,\,Da,\,\varphi$ and $Sr.$ It is indicated that the temperature increases by increasing the $\beta .$ Moreover, the concentration decreases and increases by increasing of $Sc,$ Besides, the axial velocity, and the heat transfer coefficient are increasing and decreasing by increasing $Gr$ and $\varphi ,$ respectively. In addition to, the axial pressure gradient and the mass transfer coefficient are decreasing and increasing by $Da$ and $Sr,$ respectively. For all physical quantities, we obtain the peristaltic flow in 3D overlapping and damping when the state of particle equilibrium is reached and increased. The vertical distance of the curves is greater, with most physical fields moving in peristaltic flow.

## Conclusion

The study examines the interaction of heat and mass transfer and peristaltic flow of a fractional second-grade fluid through a porous tube under low Reynolds number and long-wavelength approximation. Caputo's definition is used for differentiating the fractional derivatives. Analytical solutions are derived for temperature, concentration, axial velocity, pressure gradient, pressure rise, frictional forces, and coefficient of heat and mass transfer. The main achievement of physical parameters is illustrated. A side-by-side comparative analysis is performed to compare our findings between second-grade fluid and fractional second-grade fluid. Moreover, the fractional second-grade fluid model reduces to second-grade models for $\alpha_{1} = 1$ and classical Naiver Stokes fluid model can be deduced from this as a special case by taking $\overline{\lambda }_{1} = 0$. This provides a useful accuracy check about the correctness and validity of our results and provides a strong confidence in the presented mathematical descriptions. The major findings of the performed analysis are listed as follows:

1. The axial velocity decreases and increases with the increase of $\alpha_{1} ,\,\varphi ,\,\beta ,\,Da,\,\,M,\,\,Gr,\,\,Gn,\,\,\lambda_{1}$ and $Sc$ due to different models of second–grade fluid, fractional second-grade fluid and classical Navier–Stokes fluid, respectively.
2. The temperature increases and decreases with increasing source/sink parameter and wave amplitude.
3. The concentration decreases with the increase of both $Sr$ and $Sc.$
4. Pressure rise decrease and increase with an increase in Grashof number and Darcy number.
5. It is observed that frictional forces have an opposite behavior to that of pressure rise.
6. The value of heat and mass transfer coefficient has an oscillatory behavior due to peristalsis.
7. The existence of magnetic resonance imaging (MRI) and magnetic gadgets with a magnetic field allows for explaining the essential functions of living species. Inspired by these ideas, the current research project aims to investigate the influence of magnetic field, heat and mass transfer responses on the peristaltic pump of a fractional second-grade fluid.
8. It is found that the magnetic field effect controls the velocity and temperature of the fluid. Hence magnetic field is used in cancer therapy.
9. The results presented in this paper should prove useful for researchers in science and engineering, as well as for those working on the development of fractional second-grade fluid in a tube. Study of the phenomenon of the different physical parameters, material constant, magnetic field, and fractional parameter.

## Supplementary Information

Supplementary Information.

## Data Availability

The datasets generated and/or analysed during the current study are not publicly available due [All the required data are only with the corresponding author] but are available from the corresponding author on reasonable request.
