# Supplementary material for: Peristaltic pump with heat and mass transfer of a fractional second grade fluid through porous medium inside a tube
Source: Sci Rep. 2022 Jun 23;12:10608. doi: 10.1038/s41598-022-14773-y (PMC9225999; doi:10.1038/s41598-022-14773-y)
Supplement: Supplementary file 1 — Supplementary Information. [file 41598_2022_14773_MOESM1_ESM.docx]

**List of Symbols**

| $\overline{H}$ | The geometry of the surface |
| --- | --- |
| $a$ | The radius of the tube |
| $b$ | Wave amplitude |
| $\lambda$ | Wavelength |
| $c$ | Wave speed |
| $\overline{t}$ | Time in the fixed frame |
| $t$ | Time in the wave frame |
| $\overline{U},\overline{W}$ | Velocity components in the radial, axial directions in the fixed frame |
| $\overline{u},\overline{w}$ | Velocity components in the radial, axial directions in the wave frame |
| $\overline{P}$ | Pressure in the fixed frame |
| $\overline{p}$ | Pressure in the wave frame |
| $\rho$ | Constant density |
| $g$ | Acceleration due to gravity |
| $\sigma$ | Electrical conductivity of the fluid |
| $B_{0}$ | The intensity of the external magnetic field |
| $\alpha_{t}$ | Coefficient linear of the thermal expansion |
| $\alpha_{c}$ | Coefficient of the viscosity at constant concentration |
| $c_{p}$ | Speciﬁc heat |
| $K$ | Thermal conductivity |
| $Q_{O}$ | Heat generation coefficient |
| $D_{m}$ | Coefficient of mass diffusivity |
| $K_{T}$ | Thermal-diffusion ratio |
| $T_{m}$ | The temperature of the medium |
| $\varphi$ | Wave amplitude in non-dimensional form |
| $\theta$ | Temperature distribution |
| $\Theta$ | Concentration distribution |
| $T_{0}$ | The wall temperature of the tube |
| $C_{o}$ | The wall concentration of the tube |
| $\lambda_{1}$ | The ratio of relaxation to retardation times |
| $\alpha_{1}$ | Fractional time derivative parameter |
| $\overline{\gamma^{.} }$ | Shear rate |
| $\delta$ | Wavenumber |
| $\mu$ | The viscosity of the fluid |
| $\mathrm{Re}$ | Reynolds number |
| $\Pr$ | Prandtl number |
| $\beta$ | Heat source/sink parameter |
| $M$ | Hartmann number |
| $\Pr$ | Prandtl number |
| $Gn$ | Local concentration Grashof number |
| $Gr$ | Grashof number |
| $Da$ | Darcy number |
| $Sr$ | Soret number |
| $Sc$ | Schmidt number |
| $q$ | The volume flow rate in the wave frame |

**Appendix**

$f=\left(1+\lambda_{1}^{\alpha_{1}}D_{t}^{\alpha_{1}}\right),$ $A=\left(M^{2}+\frac{1}{Da}\right),$ $B=\left(\frac{Gr\beta}{2}-\frac{GrSrSc\beta}{2}\right),$

$$\begin{matrix} a_{1}=1-2h^{2}-\frac{f h^{2}\beta}{A^{2}}+\frac{7 h^{4}\beta}{8A}-\varphi+\frac{3\varphi^{2}}{8}+\frac{4f \log\left(-4f\right)}{A}-h^{2}\log\left(-4f\right)+\frac{3Bh^{4}\log\left(-4f\right)}{4A} \\ +\frac{4f\left(f-Ah^{2}\right)\beta\log\left(-4f\right)}{A^{3}}-\frac{4f \log\left(-4f+Ah^{2}\right)}{A}, \end{matrix}$$

$$a_{3}=-\frac{ h^{2}}{A}+\frac{4f \log\left(-4f\right)}{A^{2}}-\frac{h^{2} \log\left(-4f\right)}{A}-\frac{4f \log\left(-4f+Ah^{2}\right)}{A^{2}}+\frac{h^{2} \log\left(-4f+Ah^{2}\right)}{A}.$$
